# Gq/11-Mediated Signaling and Hypertrophy in Mice with Cardiac-Specific Transgenic Expression of Regulator of G-Protein Signaling 2

**DOI:** 10.1371/journal.pone.0040048

**Published:** 2012-07-03

**Authors:** Cindy Park-Windhol, Peng Zhang, Ming Zhu, Jialin Su, Leonard Chaves, Angel E. Maldonado, Michelle E. King, Lisa Rickey, Darragh Cullen, Ulrike Mende

**Affiliations:** 1 Cardiology Division, Cardiovascular Research Center, Rhode Island Hospital and Alpert Medical School of Brown University, Providence, Rhode Island, United States of America; 2 Department of Molecular Pharmacology, Physiology and Biotechnology, Brown University, Providence, Rhode Island, United States of America; 3 Cardiac Muscle Research Laboratory, Cardiovascular Division, Department of Medicine, Brigham and Women’s Hospital and Harvard Medical School, Boston, Massachusetts, United States of America; Ohio State University, United States of America

## Abstract

Cardiac hypertrophy is a well-established risk factor for cardiovascular morbidity and mortality. Activation of G_q/11_-mediated signaling is required for pressure overload-induced cardiomyocyte (CM) hypertrophy to develop. We previously showed that among Regulators of G protein Signaling, RGS2 selectively inhibits G_q/11_ signaling and its hypertrophic effects in isolated CM. In this study, we generated transgenic mice with CM-specific, conditional RGS2 expression (dTG) to investigate whether RGS2 overexpression can be used to attenuate G_q/11_-mediated signaling and hypertrophy *in vivo*. Transverse aortic constriction (TAC) induced a comparable rise in ventricular mass and ANF expression and corresponding hemodynamic changes in dTG compared to wild types (WT), regardless of the TAC duration (1-8 wks) and timing of RGS2 expression (from birth or adulthood). Inhibition of endothelin-1-induced G_q/11_-mediated phospholipase C β activity in ventricles and atrial appendages indicated functionality of transgenic RGS2. However, the inhibitory effect of transgenic RGS2 on G_q/11_-mediated PLCβ activation differed between ventricles and atria: (i) in sham-operated dTG mice the magnitude of the inhibitory effect was less pronounced in ventricles than in atria, and (ii) after TAC, negative regulation of G_q/11_ signaling was absent in ventricles but fully preserved in atria. Neither difference could be explained by differences in expression levels, including marked RGS2 downregulation after TAC in left ventricle and atrium. Counter-regulatory changes in other G_q/11_-regulating RGS proteins (RGS4, RGS5, RGS6) and random insertion were also excluded as potential causes. Taken together, despite ample evidence for a role of RGS2 in negatively regulating G_q/11_ signaling and hypertrophy in CM, CM-specific RGS2 overexpression in transgenic mice *in vivo* did not lead to attenuate ventricular G_q/11_-mediated signaling and hypertrophy in response to pressure overload. Furthermore, our study suggests chamber-specific differences in the regulation of RGS2 functionality and potential future utility of the new transgenic model in mitigating G_q/11_ signaling in the atria *in vivo*.

## Introduction

Myocardial growth is a response of the cardiac muscle to altered conditions of hemodynamic load. Pathological hypertrophy (e.g., in response to excess hemodynamic workload, such as in volume or pressure overload or after myocardial infarction) is a well-established risk factor for cardiovascular morbidity and mortality [1]. It is part of a remodeling response that often leads to functional decompensation and development of overt heart failure with an increased risk of sudden cardiac death.

Many signaling pathways are involved in triggering cardiac hypertrophy in response to hemodynamic stress [2]. Signaling via heterotrimeric G_q/11_ proteins is required for pressure overload hypertrophy to develop [3], [4]. G_q/11_ signaling can be negatively regulated by Regulators of G protein Signaling (RGS proteins), several of which are expressed in the heart [5]. RGS proteins negatively regulate G protein-coupled receptor signaling by accelerating signal termination via direct binding of the RGS core domain to activated GTP-bound Gα subunits [6], which leads to an increase in the intrinsic rate of Gα GTP hydrolysis [7]. In addition, RGS proteins can diminish signal production by blocking effectors from access to Gα subunits [8], [9].

The specificity of RGS proteins towards particular G proteins varies between different RGS isoforms [5]. Among the RGS proteins expressed in cardiac myocytes, the RGS2 isoform has emerged as a selective negative regulator of G_q/11_ signaling: In contrast to olfactory cells [10], RGS2 does not directly inhibit adenylate cyclase (AC) in adult ventricular myocytes [11]. RGS2 also does not inhibit muscarinic inhibitory G_i/o_-mediated cAMP regulation, which differentiates it from other G_q/11_-regulating RGS proteins in adult ventricular myocytes (such as RGS3, RGS4 and RGS5) [11]. Transgenic overexpression of RGS4 and RGS5 in the heart leads to an inhibition of pressure overload hypertrophy but with very different functional consequences (i.e., rapid decompensation and increased mortality vs. moderate improvement in contractile function, respectively) [12], [13].

Despite the presence of other G_q/11_-regulating RGS proteins, loss-of-function studies in rat ventricular myocytes in vitro (via RGS2 RNAi, [14]) and in mice in vivo (in a global RGS2 knockout model, [15]) demonstrated that endogenous RGS2 is a functionally important negative regulator of G_q/11_ signaling and hypertrophy. Gain-of-function studies showed that RGS2 overexpression in rat ventricular myocytes inhibits G_q/11_-mediated signaling and hypertrophy development in vitro [11], [16].

The goal of the present study was to test whether cardiac-specific transgenic overexpression of RGS2 in mice can be used to attenuate the hypertrophic response to pressure overload by inhibiting G_q/11_-mediated signal transduction *in vivo*. To that end, we generated a mouse model with cardiac-specific RGS2 expression, examined its hypertrophic response to transverse aortic constriction (TAC) and investigated the effect of transgenic RGS2 expression on endothelin-1 (ET-1) induced activation of phospholipase C β (PLCβ), the immediate downstream target of G_q/11_ proteins. Since transgenic phenotypes can vary depending on the timing of transgene expression [17], [18], we utilized a conditional Tet-off binary model [19] to investigate the effects of RGS2 when it was expressed conditionally as of adulthood or constitutively, based on the intrinsic characteristics of the α-myosin heavy chain (MHC) promoter used to drive transgene expression.

## Results

### Generation of the RGS2 Transgenic Mouse Model

**Figure 1:**
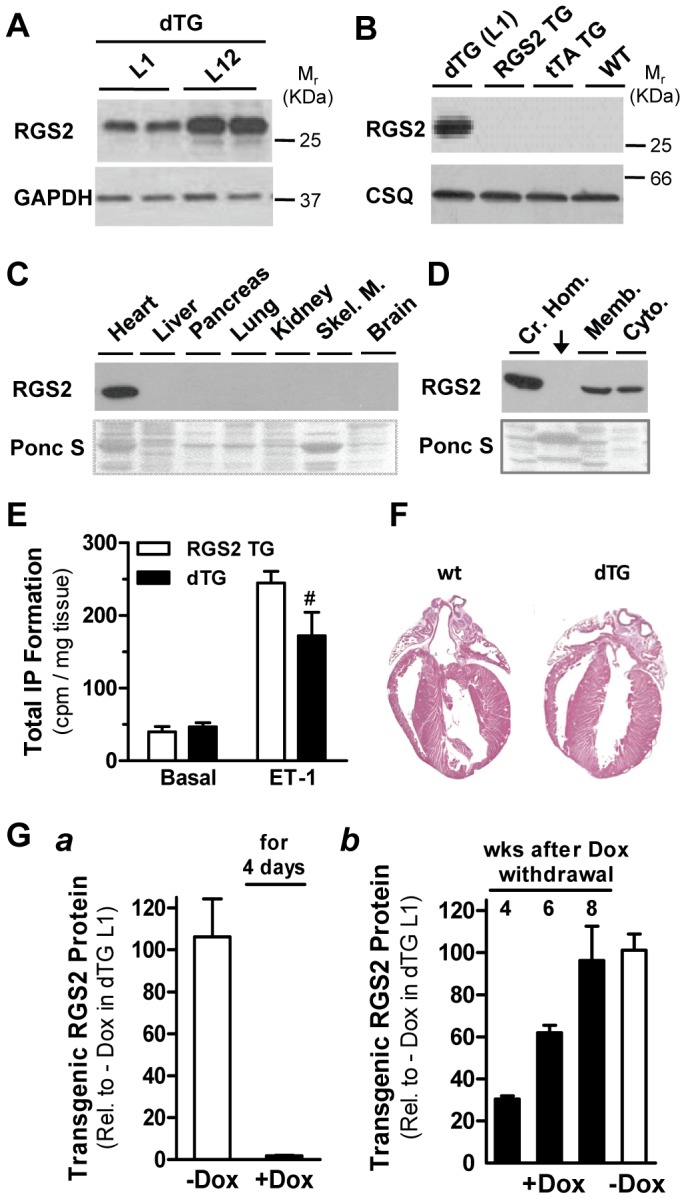
Transgenic RGS2 Protein is expressed in a cardiac-specific, tTA-dependent, Dox-regulatable manner and is functionally active. [**A**] Representative Western blot of crude ventricular homogenates (10 µg/lane) from 3-months old double transgenes (dTG) of two independent transgenic RGS2 lines (L1, L12). The blot was probed with antibodies recognizing RGS2 and GAPDH, which was used as loading control. [**B**–**D**] Western blots of crude ventricular homogenates from indicated genotypes obtained from Line 1 matings (B), of crude organ homogenates from dTG mice only (C), and membrane and cytosolic fractions from dTG ventricles (D). Each blot was probed with an anti-FLAG antibody. Calsequestrin expression and Ponceau S stain were used as controls (panels B and C/D, respectively). The arrow in panel D denotes a lane loaded with a molecular weight marker. [**E**] Total inositol phosphate (IP) formation in ventricles from RGS2 TG (open bars, n = 3) and dTG mice (n = 4 each; closed bars). Freshly dissected tissue pieces were incubated for 30 min in the absence (basal) or presence of endothelin-1 (ET-1, 100 nM). # P<0.05 dTG vs RGS2 TG. [**F**] H&E stained four-chamber sections of hearts from a 12-week-old wild-type (WT) and dTG (L1) mouse. [**G**] Quantitative Western blot analysis of crude ventricular homogenates from dTG mice: (a) RGS2 protein suppression within 4 days after Doxycycline (Dox, 100 mg/kg) administration. (b) RGS2 re-expression in mice, which had received Dox (100 mg/kg) from gestation until 3 weeks of age, after indicated weeks of withdrawal from Dox (closed bars, n = 4 each); untreated 11 week-old dTG controls (- Dox, open bar, n = 5) are shown for comparison.

Using a binary tetracycline-based, murine α-MHC promoter-driven system [19], we generated two independent mouse models (Figure 1A) with cardiac-specific and tetracycline-regulatable expression of N-terminally FLAG-tagged RGS2. Most experiments were conducted in Line 1 (L1) mice; where indicated select findings were confirmed in a second line (L12). Figure 1A illustrates RGS2 protein expression levels after crossbreeding each RGS2 transgenic line with tTA TG mice. At 3 weeks of age and in the absence of Dox (i.e., when tTA can bind to the Tet operon embedded in the α-MHC promoter of the responder line [19]), transgenic RGS2 protein was expressed in crude ventricular homogenates from double RGS2/tTA transgenic mice (dTG) only; no FLAG-RGS2 was detected in RGS2 TG (or tTA TG and WT), indicating that the expression of RGS2 is strictly tTA-dependent (Figure 1B). Expression of transgenic RGS2 protein was cardiac-specific (Figure 1C) and equally detectable in membrane and cytosolic fractions (Figure 1D).

We previously demonstrated that a FLAG tag at the N-terminus of RGS2 does not affect the inhibitory effect of RGS2 on G_q/11_-mediated signaling [20]. Functionality of transgenic RGS2 was confirmed by inhibition of endothelin-1 (ET-1)-induced PLCβ activity in ventricles from RGS2-expressing dTG mice compared to single RGS2 TG mice (Figure 1E). To that end, we measured total inositol phosphate (IP) formation as a reflection of PLCβ activity, the immediate downstream target of G_q/11_. RGS2-expressing dTG mice were healthy and showed no changes in cardiac weight (data not shown) or morphology (Figure 1F) under baseline conditions.

Comparing different Dox concentrations (100 to 1000 mg/kg), we established the minimal Dox dosage (100 mg/kg) to fully suppress RGS2 expression (shown after 4 days in Figure 1G, panel a). In mice treated with Dox from gestation until 3 weeks of age, RGS2 was fully re-expressed within 8 weeks after Dox withdrawal compared to untreated dTG mice of the same age (panel b).

Taken together, the new binary RGS2 transgenic mouse model showed the expected characteristics with regard to cardiac specificity and Dox sensitivity of RGS2 expression and its functionality. We selected WT mice (gender-matched littermates) as controls for dTG mice after pilot experiments confirmed that single tTA and single RGS2 transgenic mice had similar cardiac weights compared to WT mice both under basal conditions and in response to pressure overload (data not shown).

### Effect of RGS2 Expression in vivo on G_q/11_-mediated Hypertrophy Development and LV Function

**Figure 2:**
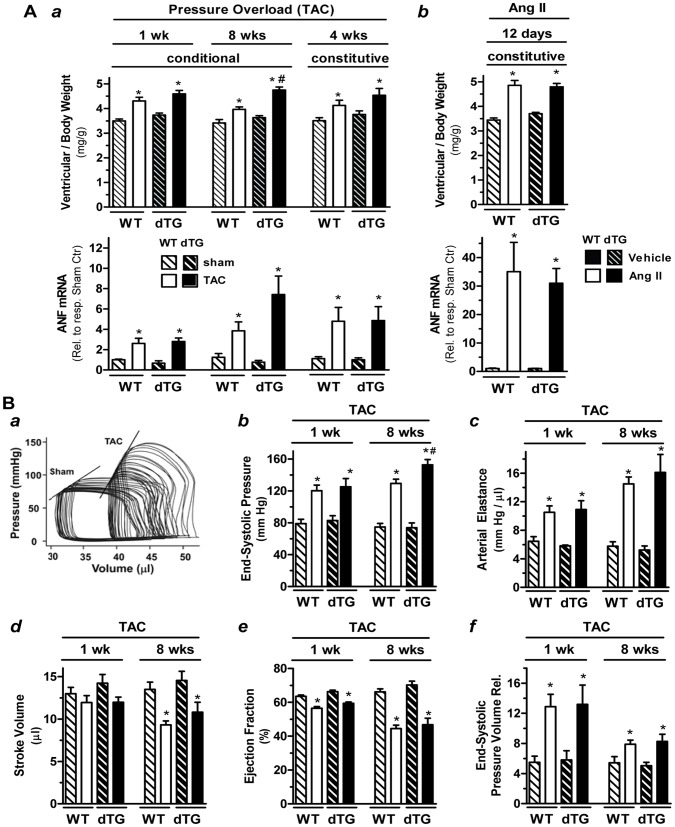
dTG and WT mice have comparable hypertrophic and functional responses to TAC and Ang II. [**A**] Hypertrophic response to pressure overload (*panel a*) and Ang II infusion (*panel b*): Ventricular weight (normalized to body weight, *top panel*) and ANF mRNA (normalized to 18S and expressed relative to sham controls, *bottom panel*) in wild-type mice (WT) and double transgenic mice (dTG, L1) with conditional or constitutive RGS2 transgene expression after transverse aortic constriction (TAC, n = 10–18 WT and n = 11–15 dTG) or Ang II infusion (n = 9 each) for indicated durations (*solid bars*). Animals subjected to sham operation (n = 9–15 WT and n = 7–9 dTG) or vehicle (saline, n = 5–6 each) were used for comparison (*cross-hatched bars*). ANF expression was assessed at least in half of the animals in each group. * *P*<0.05 TAC vs sham (a) or Ang II vs vehicle (b); # *P*<0.05 dTG vs WT. [**B**] *In vivo* hemodynamic assessment of left ventricular function: (a) Representative pressure volume (PV) recordings during preload reduction via inferior vena cava compression in WT mice that had been subjected to TAC or sham operation 4 wks earlier. The traces show raw data before subtraction of volume from parallel conduction. The solid lines indicate end-systolic pressure volume relationship (ESPVR). (b) Group data obtained from PV loop analyses for indicated parameters in WT and dTG mice with conditional RGS2 expression (L1) after TAC for 1 wk (n = 11 WT and n = 8 dTG) or 8 wks (n = 13–15 WT and n = 8–9 dTG) compared to respective sham controls (1 wk: n = 9 WT and n = 5 dTG; 8 wks: n = 7–8 WT and n = 5–6 dTG). * *P*<0.05 TAC vs Sham; # *P*<0.05 dTG vs wt.

We determined the effect of both conditional and constitutive transgenic RGS2 overexpression on hypertrophy development in response to pressure overload and angiotensin II (Ang II) infusion, which activates G_q/11_-coupled AT_1_ receptors directly. As expected, in WT mice TAC - when applied for 1, 4 and 8 weeks - induced characteristic increases in ventricular weight/body weight ratios (Figure 2A, panel a, top) and ANF mRNA expression (bottom). The TAC-induced changes in dTG, irrespective of TAC duration and the timing of transgenic RGS2 expression, were not attenuated compared to WT as hypothesized but comparable (or slightly enhanced after 8 wks TAC only). In response to Ang II infusion for 12 days (Figure 2A, panel b), the hypertrophic response in WT was more pronounced than that observed after TAC (141% vs. 123% increase in VW/BW; 35-fold vs. 3-fold increase in ANF expression). Again, dTG mice had a comparable response to WT mice. Taken together, these data demonstrate that G_q/11_-mediated hypertrophy development is not attenuated in mice with cardiomyocyte-specific RGS2 overexpression *in vivo.* Similar results were observed in mice from RGS2 transgenic mice from L12 (constitutive RGS2 expression, 10 day TAC, data not shown), which strongly suggests that random insertion of the transgene into the genome does not contribute to the observed phenotype.

We next assessed cardiac *in vivo* hemodynamics in WT and dTG mice (L1) 1 wk or 8 wks after sham operation or TAC. Mean heart rates during the measurements ranged from 470 to 542 bpm in WT and dTG mice with no differences between sham and TAC (data not shown). Representative PV loop recordings from WT mice in response to preload reduction are shown in Figure 2B (panel a). WT mice showed characteristic increases in end-systolic pressure (panel b) and arterial elastance (which reflects total ventricular afterload, panel c), a progressive reduction in stroke volume and ejection fraction (panels d/e) and a steepening of the end-systolic pressure volume relationship (indicating increased contractility after 1 wk TAC) that was comparatively blunted after 8 wks (panel f). In dTG mice, both systolic and diastolic functions were comparable to WT mice under baseline conditions and in response to short- and long-term TAC. The only exception was a slight increase in end-systolic pressure after 8 wks TAC that correlated with a modest increase in pressure gradient (data not shown). These data demonstrate that regardless of the duration of TAC, functional responses to pressure overload were not altered in RGS2-expressing mice compared to WT.

### Effect of RGS2 Expression in vivo on G_q/11_-mediated PLCβ Activation in Response to TAC

**Figure 3:**
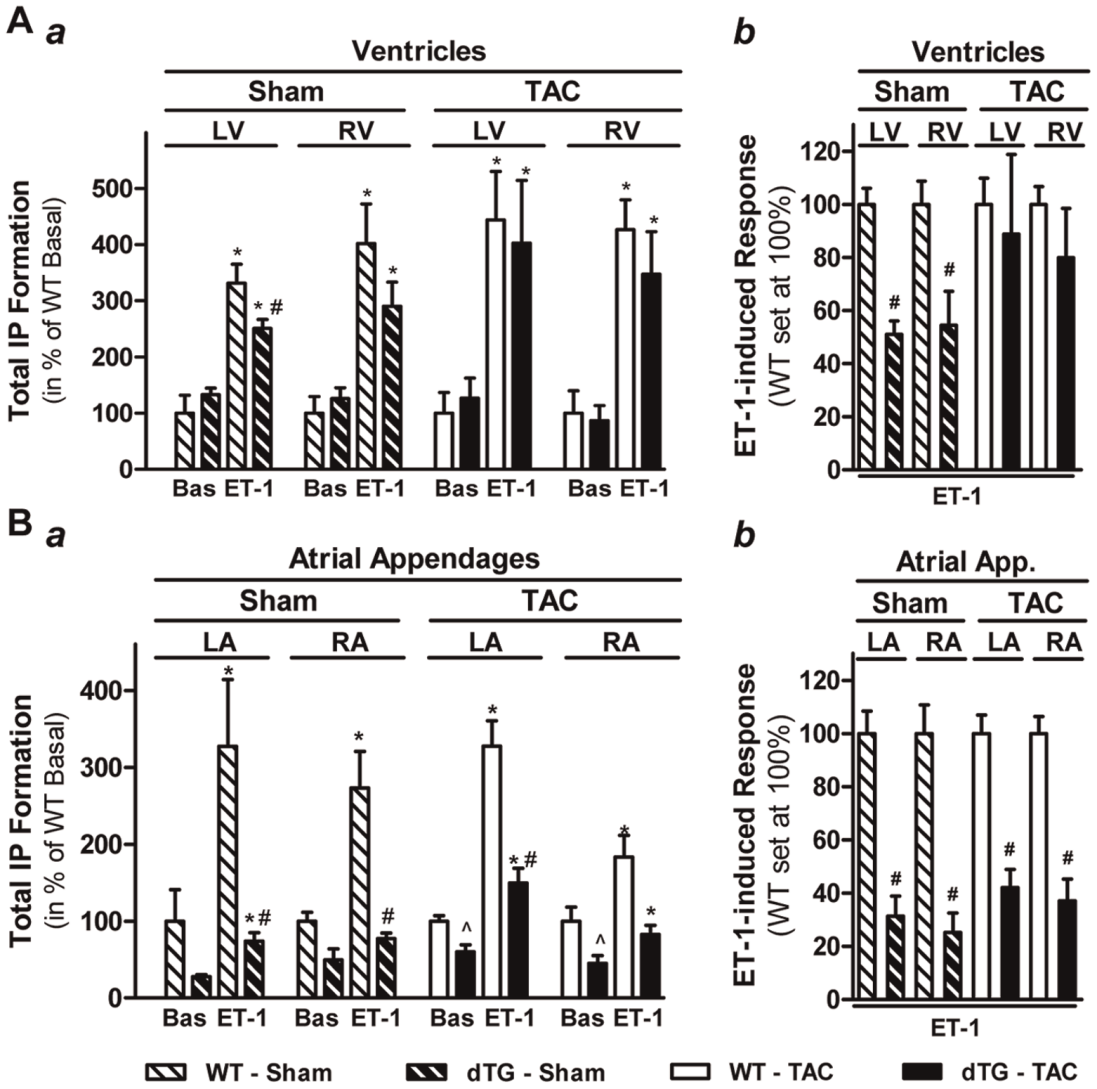
Transgenic RGS2 expression *in vivo* differentially inhibits G_q/11_-mediated PLCβ activation in ventricles and atrial appendages. Basal (Bas) and Endothelin-1 (ET-1)-induced (100 nM, 30 min) total inositol phosphate (IP) formation in left and right ventricles [**A**] as well as left and right atrial appendages [**B**] from WT mice (*open bars*) and dTG mice (L1, constitutive RGS2 expression, *closed bars*) 6 wks after sham-operation (*cross-hatched bars,* n = 5 WT and dTG each) or TAC (*solid bars*, n = 4 WT and n = 6 dTG). Data are expressed in % of basal values in WT for each region (panel A: 18–25 cpm/mg tissue for LV and RV after sham or TAC; panel B: 100-140 and 55–85 cpm/mg for LA and RA after sham or TAC, respectively). *P<0.05 ET-1 vs basal; #*P*<0.05 dTG vs WT (ET-1 effect); ^P<0.05 dTG vs WT (basal). The right panels (b) depict the inhibition of ET-1-induced IP formation (i.e., difference in IP formation in the presence or absence of ET-1 in the respective panel a) in indicated regions of dTG mice. Data are expressed in % of the ET-1-induced response in WT (set at 100%, not shown). #*P*<0.05 dTG vs WT.

Next, we determined whether transgenic RGS2 was still functionally active in inhibiting cardiac G_q/11_ signaling in dTG ventricles after TAC (Figure 3A, panel a). To measure basal and G_q/11_-coupled receptor-induced PLCβ activity in left and right ventricles from dTG and WT mice 6 wks after they had been subjected to TAC (or sham operation), we incubated small tissue pieces from both chambers for 30 min in the absence or presence of endothelin-1 (100 nM). ET-1 caused a comparable increase in PLCβ activity in both ventricles in WT, with no major differences between sham and TAC operation. In both LV and RV, transgenic RGS2 expression in sham-operated dTG mice did not alter basal PLCβ activity but inhibited ET-1-induced PLCβ activity. Importantly, the inhibitory effect of RGS2 was no longer observed after TAC.

Since α-MHC- promoter driven transgenes are also expressed in atrial tissue, we also assessed basal and ET-1-induced PLCβ activity in atrial appendages from the same animals (Figure 3B, panel a). While basal activity was slightly higher in WT atrial appendages than ventricles (see legend for Figure 3), the stimulatory effect of ET-1 was overall comparable. In dTG atrial appendages, we observed marked inhibition of ET-1-induced PLCβ activation in sham-operated dTG mice that in contrast to the ventricles was preserved after TAC. Basal PLCβ activity was slightly diminished in dTG atrial tissue but not ventricles. Panel b in Figures 3A and 3B depict the inhibitory effect of transgenic RGS2 expression on ET-1-induced PLCβ activity in ventricles and atrial appendages, respectively, independent from the basal activity. We calculated the difference in ET-1-induced IP formation and basal IP formation in dTG for each region both after sham and TAC and expressed it relative to the respective response in WT, which was set as 100%. In sham-operated RGS2-expressing ventricles, ET-1-induced PLCβ activation was inhibited to 51±5% (LV) and 54±13% (RV) of WT (P<0.05). After TAC, the inhibitory effect was marginal and not significant (89±30% and 80±18% of WT, respectively). In LA and RA, PLCβ inhibition was more pronounced than in ventricles (31±8% and 25±7% of WT, respectively) and preserved after TAC (42±7% and 37±8% of WT). Taken together, the response of ventricles and atrial appendages to transgenic RGS2 expression differed with regard to (i) its effect on basal PLCβ activity (reduced in atrial appendages but not ventricles), (ii) the extent of inhibition of G_q/11_-mediated PLCβ activation (more pronounced in atrial appendages than ventricles) and (iii) preservation of the inhibitory RGS2 effect after TAC (in atrial appendages but not ventricles).

### Expression of RGS2 (and Other RGS Proteins) and their Regulation by TAC and Ang II

**Figure 4:**
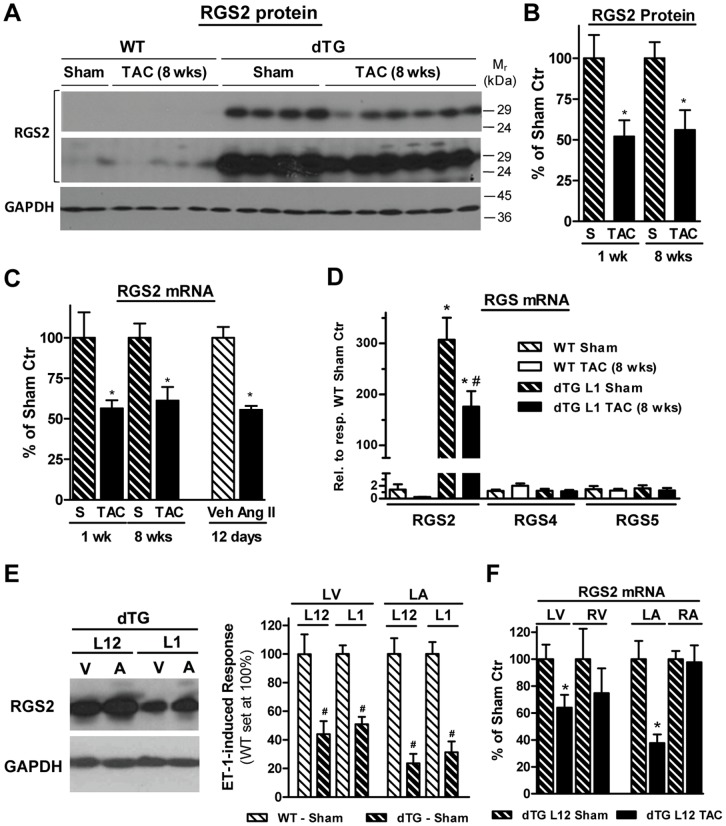
TAC and Ang II induce chamber-specific down-regulation of transgenic RGS2 protein and mRNA. [**A**] Representative Western blot of crude ventricular homogenates (60 µg/lane) from WT and dTG (L1) ventricles after sham or 8 wk TAC, which were probed with antibodies recognizing RGS2 (1 and 10 sec exposure times) and GAPDH. [**B**] Ventricular RGS2 protein downregulation in dTG mice (L1, conditional transgene expression) after TAC for 1 wk (n = 9) or 8 wks (n = 15) compared to respective sham controls (n = 7–9). * *P*<0.05 TAC vs Sham. [**C**] Ventricular RGS2 mRNA downregulation in dTG mice (L1, conditional transgene expression) after TAC for 1 wk (n = 6) or 8 wks (n = 12) compared to respective sham controls (n = 6–8). The effect of Ang II infusion (n = 4) compared to vehicle controls (n = 2) for 12 days is also shown. * *P*<0.05 TAC vs Sham or Ang II vs vehicle respectively. [**D**] Ventricular RGS2, RGS4 and RGS5 mRNA expression in WT and dTG (L1) mice 8 wks after TAC or sham operation. Real-time PCR data normalized to 18S are expressed relative to respective WT sham (n = 7–10 in each group, except for n = 3 for RGS2 in WT). [**E**] *Left:* Representative Western blot of crude homogenates (30 µg/lane) of L12 and L1 dTG ventricles (V) and atrial appendages (A) probed with antibodies recognizing RGS2 and GAPDH. *Right:* Inhibition of ET-1-induced IP formation in left atrial appendages (LA) and ventricles (LV) from sham-operated L12 (n = 6–7) and L1 (n = 5) dTG. Data are expressed in % of the ET-1-induced response in WT (set at 100%, not shown). # *P*<0.05 dTG vs WT (set at 100%). [**F**] RGS2 mRNA expression in left and right ventricles and atrial appendages from dTG L12 mice (constitutive transgene expression) 10 days after TAC compared to respective sham controls (n = 5–7). * *P*<0.05 TAC vs Sham.

We examined RGS2 protein and mRNA expression in dTG mice from L1 (Figure 4A–D) and L12 (Figure 4E/F) as a potential explanation for the lack of G_q/11_ signal inhibition and hypertrophy in dTG ventricles as well as for the chamber-specific differences we observed in the effectiveness of RGS2-mediated inhibition of G_q/11_ signaling. First, we addressed in L1 mice whether RGS2 expression is altered in response to TAC or Ang II infusion. As shown by a representative Western blot (Figure 4A) and group data (Figure 4B), RGS2 protein was markedly down-regulated in LV crude homogenates as early as 1 wk after TAC, which persisted until 8 wks (52±17% and 56±12% compared to sham Ctr, respectively). This effect was accompanied by corresponding decreases in mRNA expression at both time points (Figure 4C). Similar RGS2 down-regulation was observed after Ang II infusion (Figure 4C). Importantly, despite the TAC-induced decrease in RGS2 expression in dTG, the overall expression level of RGS2 in dTG was still markedly enhanced over endogenous RGS2 in WT on both protein and mRNA level (Figure 4A/D).

Figure 4E (left) illustrates that in dTG L12 mice RGS2 protein levels were higher in both ventricles (see also Figure 1A) and atria compared to L1 and that transgenic RGS2 expression was higher in the atria than ventricles in both lines. These differences in expression allowed us to indirectly address whether regional differences in transgene expression could account for the differences in functional activity of RGS2 we observed in the different cardiac regions (atria > ventricles, see Figure 3). This does not seem to be the case, because in spite of greater RGS2 expression, the inhibitory effect on ET-1 induced PLCβ activation in L12 dTG LV and LA was comparable to that in L1 dTG (Figure 4E, right panel). Figure 4F shows the effect of TAC on RGS2 mRNA expression in each chamber from L12 dTG mice. RGS2 down-regulation was observed in LV as well as in LA. In contrast, RGS2 expression in RA was not altered. Similar results were observed in atrial appendages from L1 dTG mice (not shown). RGS2 expression in the RV was more variable in both sham and TAC mice and did not reach statistical significance.

Taken together, RGS2 protein and mRNA in dTG are marked down-regulated in LV and LA after TAC and Ang II infusion. The reduction in RGS2 expression in response to the hypertrophic stimulus was rapid (within 1 wk) and persistent (until 8 wks). The remaining levels were still significantly enhanced compared to non-transgenic controls. Region-specific differences in RGS2 expression levels and its regulation by TAC were observed.

We also explored whether transgenic RGS2 expression affects the expression of other RGS proteins. RGS4 and RGS5 mRNA levels were comparable in ventricles from dTG and WT mice (after sham operation or TAC, Figure 4D). In dTG and WT atrial appendages (data not shown), we observed a slight reduction in RGS5 mRNA and comparable levels of RGS4 and RGS6, which was examined as well in light of its central role in atria [21], [22].

## Discussion

Hypertrophy is a well-established risk factor for cardiovascular morbidity and mortality [23], [24]. While the initial hypertrophic response to hemodynamic stress was long viewed as a necessary compensatory effect, it has been shown that hypertrophy is not necessarily required to preserve heart function [25]–[27]. Many different strategies have been pursued to inhibit and/or reverse pathological hypertrophy [2], [28], [29]. It is believed that leveraging endogenous negative regulators of hypertrophy may represent a promising strategy, because it can mimic negative feedback mechanisms that are often central for maintaining cellular homeostasis [30], [31]. The present study was designed to test whether overexpression of RGS2 in cardiomyocytes *in vivo* can be used as a strategy to inhibit pressure overload hypertrophy in mice by modulating the finely tuned G_q/11_ signaling pathway. Using a novel mouse model with cardiomyocyte-specific and inducible transgenic RGS2 expression, our study suggests that this may not be the case for reasons that are not yet fully understood. We demonstrate that transgenic RGS2 is highly functional in negatively regulating G_q/11_ signaling in atrial appendages and to a lesser extent in ventricles. In ventricles (but not atrial appendages), RGS2-mediated modulation of G_q/11_ signaling was absent after TAC, which may account for the lack of anti-hypertrophic effect. Importantly, TAC-induced RGS2 expression changes (down-regulation) do not seem to underlie the functional differences we observed between atria and ventricles. Similar results were observed in two independent transgenic lines, making it highly unlikely that random insertion contributed to the phenotype in this model. Changes in the expression of other RGS proteins are also unlikely to play a role.

We used a well characterized binary Tet-off system [19] to generate a new mouse model that shows characteristic cardiac-specific, tTA-dependent and doxycycline-regulatable RGS2 transgene expression. It has been reported that depending on the expression level, tTA expression itself can have phenotypic effects in the myocardium [19], [32]. In this study, crossbreeding was performed using tTA transgenic mice with a very low level of tTA expression, which is sufficient to activate expression of the responder gene but does not alter cardiac weight or function [19]. Since Dox exposure can also affect the hypertrophic response in mice [33], [34], we compared animals that had been subjected to the same Dox treatment.

Transgenic RGS2 was robustly overexpressed in heterozygous dTG mice, but it was reduced on both mRNA and protein levels after TAC. The extent of down-regulation in our model was substantial (up to 48%) and was detected as early as 1 wk after TAC and maintained until 8 wks after TAC. The time course and magnitude of the RGS2 transgene reduction we observed is not without precedent. For example, a 40% reduction in α-MHC driven β_2_-receptor transgene expression was reported 1 and 3 wks after TAC, with further reduction (by 70%) upon prolonged TAC (8 wks), which correlated with a progression in hypertrophy development [35]. In our model, we did not observe a further increase in the TAC-induced rise in ventricular weight or ANF expression between 1 and 8 wks, which is characteristic for FVB mice [36], [37] and different from C57BL/6 mice [37], the strain used by the other group [35].

Transcriptional regulation could be involved in RGS2 down-regulation after TAC in dTG mice, since RGS2 mRNA and protein levels were affected to a similar extent. Changes in RGS2 mRNA stability may contribute as well, since endogenous RGS2 mRNA in WT mice, which is driven by its own promoter, is also subject to TAC-induced down-regulation in ventricles [14] (see also Figure 4D). Additional changes in protein stability cannot be excluded.

Little is known from other studies about region-specific differences in α-MHC promoter driven transgene expression and its regulation by experimental *in vivo* stimuli. We show differences in RGS2 transgene expression (greater in atria than in ventricles) as well as its regulation by TAC (marked downregulation in both LV and LA, with little and no effects in RV and RA, respectively). Our findings have potentially important implications for other studies that use the α-MHC promoter to drive transgene expression. While identifying the underlying mechanisms is beyond the scope of this investigation, our study points to the importance of evaluating transgene expression in all regions of the heart as well as in response to experimental stimuli, since chamber-specific differences and regulation of transgene expression may contribute to the phenotype.

RGS2 is generally believed to be selective in negatively regulating G_q/11_ in the heart, which has been attributed to the geometry of its Gα-binding pocket and three evolutionary highly conserved amino acids (reviewed in [5]). In some cell types and under certain experimental conditions, RGS2 has been shown to suppress AC directly [10], [38] and to inhibit G_i/o_ signaling [39]. In adult rat ventricular myocytes, RGS2 overexpression does not affect forskolin- or isoproterenol-induced cAMP generation [11], indicating that neither direct nor indirect RGS2-induced AC regulation plays a major role in differentiated myocytes. RGS2 also does not regulate muscarinic G_i/o_-mediated inhibition of cAMP in adult ventricular myocytes [11]. A potential role as terminator of β_2_-receptor-coupled G_i_ signaling was recently proposed for cultured rat ventricular myocytes [40].

We previously demonstrated that the presence of a FLAG epitope at the N-terminus of RGS2 does not affect its ability to negatively regulate G_q/11_-mediated IP formation [20]. We also showed that overexpression of FLAG-tagged RGS2 in neonatal and adult ventricular myocytes attenuated the stimulatory effects of ET-1 and phenylephrine on IP formation [11]. In the present study, we show that transgenic RGS2 expression markedly inhibited ET-1-induced IP formation compared to controls in atrial appendages of dTG mice, indicating that transgenic RGS2 at the level expressed in our model is highly functional *in vivo.* Effective inhibition of G_q/11_ signaling by transgenic RGS2 expression in LA and RA was observed both under basal conditions (sham controls) and after TAC, when transgenic RGS2 expression was markedly down-regulated in LA (but not in RA). Conversely, further enhanced RGS2 expression (as seen in dTG L12 mice) did not further increase the inhibitory effect of RGS2 on G_q/11_ signaling (compared to L1 dTG). Taken together, these findings suggest RGS2 in our model is expressed in excess, allowing for a “reserve” that seems to ensure effective G_q/11_ signaling inhibition after TAC-induced RGS2 downregulation in LA. Given the level of RGS2 overexpression in our mouse lines, this is not surprising.

In dTG ventricles, the inhibitory effect of transgenic RGS2 was less pronounced than in atrial appendages under sham conditions. It is unlikely that this is due to lower RGS2 expression in ventricles than in atria, because the inhibitory effect was not enhanced in L12 dTG ventricles, in which ventricular RGS2 expression levels was comparable to atrial expression level in L1 mice. Importantly, after TAC, the inhibitory effect of RGS2 in ventricles was markedly blunted and not statistically significant. Counter-regulatory changes in ventricular expression of other G_q/11_-regulating RGS proteins were excluded as potential explanation. While some highly expressed proteins can be non-functional due to aberrant location, insufficient posttranslational modifications or other causes, this does not appear to be the case in our model because of the marked inhibition of G_q/11_ signaling we observed in atrial appendages with similar expression levels as in ventricles.

The lack of inhibitory effect on G_q/11_ signaling in the ventricles after prolonged TAC was associated with (and likely contributes to) the lack of anti-hypertrophic effect observed in dTG mice expressing RGS2. Importantly, irrespective of the duration of TAC (acute vs. prolonged) and the timing of RGS2 expression (conditional vs. constitutive), the hypertrophic response to pressure overload was not attenuated in dTG mice. Although TAC-induced pressure overload effects are largely mediated by G_q/11_ [3], [4], we also determined the effect of Ang II, which exerts its effects primarily via G_q/11_-receptor coupled AT_1_ signaling. Differential inhibitory effects on Ang II- and TAC-induced hypertrophy (abrogated vs. no change, respectively) have been reported in other mouse models (e.g., [41]). In our model, we observed a comparable increase in LV mass and ANF induction between dTG and WT in response to Ang II infusion.

Our findings are surprising in light of the fact that endogenous RGS2 protein is a well recognized, functionally important negative regulator of G_q/11_ signaling and hypertrophy in cardiomyocytes. This has been shown *in vitro* using RNA interference in cultured ventricular cardiac myocytes [14] as well as *in vivo* in mice with a global RGS2 knockdown [15]. The latter study showed that endogenous RGS2 is required for inhibiting the Gα_q/11_/PLCβ pathway, particularly in the early compensatory phase of pressure overload (within 1 week), because its absence was associated with rapid decompensation and increased mortality.

We and others previously demonstrated that transgenic RGS2 overexpression in ventricular myocytes is highly effective in selectively mitigating the G_q/11_-mediated PLCβ activation and the resulting hypertrophic response [11], [16]. At the levels expressed in our model, RGS2 was highly functional in the atrial appendages, but much less active in the ventricles under normal conditions and even further reduced after TAC. The reasons are not yet understood. Differences between atrial and ventricular RGS2 expression levels do not appear to be the explanation. Our findings point towards the possibility that RGS2 function may be differentially regulated in atria and ventricles *in vivo*, which could include differences in posttranslational modifications that can modulate the effectiveness of RGS2 in exerting regulatory effects on GPCR signaling (such as phosphorylation [42], [43] and palmitoylation [44], [45]). Beyond the scope of the present study, our observations warrant further investigations into potential differences in RGS2 signaling, its regulation and underlying mechanisms between atrial and ventricular myocytes, which are known to differ in structure, function, gene expression and their responses to stimuli (e.g., [46], [47]).

Since transgenic overexpression of RGS2 was associated with highly effective inhibition of G_q/11_ signaling in the atria, the new model should be useful for future studies aimed at advancing understanding of atrial signaling as well as mitigating G_q/11_ signaling in the atrium for potential therapeutic purposes. Targeting G_q/11_ signaling in the atria was recently proposed as a therapeutic strategy to prevent atrial structural and electrical remodeling and atrial fibrillation [48]. This notion is based on evidence from both animal models [49], [50] and humans [51], [52] that implicate enhanced G_q/11_ signaling activation in the pathogenesis of atrial arrhythmogenic remodeling.

## Materials and Methods

### Ethics Statement

All animal procedures were approved by the Institutional Animal Care and Use Committees of Rhode Island Hospital (protocol # 324-05, 197-08, 117-11) and Brown University (protocol # 25-07,44-08, 0903027,1003012) and performed in strict accordance with the recommendations of the Guide for the Care and Use of Laboratory Animals of the National Institutes of Health. All surgeries were performed under indicated anesthesia, and all efforts were made to minimize suffering.

### Generation of RGS2 Transgenic Mouse Model

The transgenic construct was generated by PCR amplification of cDNA encoding mouse RGS2 with a primer pair that introduced restriction sites and an N-terminal FLAG epitope 5′-ACGCGTCGACATGGACTACAAGGACGACGATGACAAG-3′ and 5′-CCCAAGCTTTCATGTAGCATGGGGCTCCGT-3′). The resulting product was subcloned via Sal I and Hind III restriction digest and ligation into a pBS II SK(+) expression vector containing an attenuated α-MHC promoter that can be inducibly regulated by tetracycline-controlled Transactivator (tTA, [19]). After restriction mapping and nucleotide sequencing, a linear 7.1-kb DNA fragment was released with Not I, gel purified (Qiaex II, Qiagen, Valencia, CA) and microinjected into pronuclei of fertilized FVB mouse oocytes and implanted in pseudopregnant females by the Transgenic Core Facilities of Harvard Medical School and Cincinatti Children’s Hospital. Heterozygous RGS2 transgenic mice (RGS2 TG) mice were crossed with heterozygous, α-MHC-promoter driven transgenic mice expressing a tTA [19]. Genotyping to distinguish wild-type (WT), RGS2 TG, tTA TG and RGS2/tTA double transgenic (dTG) mice was performed using primer pairs that are specific for transgenic RGS2 (5′-CTCCCCCATAAGAGTTTGAGTCG-3′ and 5′- TGAAGCAGCCACTTGTAGC-3′), tTA (5′-TGGGAGAGCCATAGGCTACGGTGTA-3′ and 5′-CATCGCGATGACTTAGTAAAGCACA-3′) and calsequestrin (5′-TCTTGCTCTGGGATTTCCCTACTGC-3′ and 5′CTTCCCTTTCGTGGAATGTTGATAG-3′) as positive control.

In experiments with conditional RGS2 expression, mice received doxycycline (Dox) via food pellets (100 mg/kg, PharmaServ Inc., Framingham, MA) from gestation until 3 weeks of age to prevent RGS2 transgene expression during embryonic and postnatal development (see Fig. 1G, panel a), followed by Dox withdrawal for at least 8 weeks to allow re-expression of RGS2 transgene expression (see Fig. 1G, panel b) before experiments were performed. WT mice that were subjected to the same Dox treatment served as controls. In experiments with constitutive RGS2 expression, mice did not receive any Dox.

### Histology

Hearts were fixed in 4% neutral-buffered formalin, bisected in a plane that exposed both atria and ventricles, and embedded in paraffin. Sections (5 µm) were stained with the hematoxylin and eosin.

### Mouse Surgery and Drug Treatment

Pressure overload was induced by transverse aortic constriction (TAC) as described [53] in 12-15 week old mice that were anesthetized with pentobarbital (50–70 mg/kg). The body temperature was maintained throughout the surgical procedure at 37±0.5°C via a warm water blanket and a heat lamp controlled by a TCAT-2AC Animal Temperature Controller. After endotracheal intubation, the mice were connected to a rodent ventilator (Harvard Apparatus, MiniVent Type 845, Holliston, MA). Lidocaine (2 mg/kg) was provided in the surgical area as local anesthetic. An incision was made in the chest wall at the third intercostal space and a murine rib spreader (a modified Alms Retractor 2 ¾ inches) was inserted to allow access to the thoracic cavity. A 7-0 silk suture was applied around the transverse aorta and a 27-gauge needle. After careful removal of the needle, the rib spreader was closed, and the lungs reinflated prior to each layer of closure of the chest wall with 4-0 vicril. Sham controls underwent the same procedure without aortic constriction. Alzet osmotic minipumps (no. 2002; Alza Corp, Mountain View, CA) containing either angiotensin II (Ang II, 1.46 mg/kg · day) or saline were surgically implanted subcutaneously between the shoulder blades under 1–2% isoflurane anesthesia. All mice received 0.4% KCl post surgery as a standard regimen.

### *In vivo* Hemodynamic Measurements

Left ventricular function was assessed *in vivo* using pressure-volume (PV) conductance catheters and a closed chest approach as described [54], [55]. Mice were anesthetized with 1–2% isoflurane and body temperature was closely monitored and maintained as described above. Following tracheotomy, mice were ventilated with 6 ml/kg (animal mass) tidal volume; the respiration rate (min^−1^) was calculated as 53.3 × (animal mass, kg)^−0^.^26^. A Mikro-Tip® PV catheter (PVR-1045, 1F, Millar Instruments Inc., Houston, TX) that had been calibrated according to the manufacturer’s instructions was inserted into the left ventricle through the right carotid artery and aortic valves. In addition, the right jugular vein was cannulated with a P10 tube for hypertonic saline bolus (parallel conduction calibration). Data was acquired using MPVS Ultra (Millar Instruments Inc.) and PowerLab 16/30 with LabChart 7 Pro software (ADInstrument, Inc., Colorado Springs, CO). At a sampling rate of 1 kHz, data were recorded both at steady state and during transient reduction of venous return (compression of the inferior vena cava by applying abdominal pressure adjacent to the diaphragm with a cotton Q-tip). At the end of recordings, pressure gradients between the left and right carotid arteries were measured by insertion of a second catheter into the left carotid artery. Data were stored and analyzed with LabChart 7 and PVAN Ultra softwares by investigators blinded to genotype. PV relationships at the end of systole and diastole were derived from recordings after inferior vena cava occlusion.

### Analysis of Protein Expression

Snap-frozen cardiac tissue was homogenized, separated on 4–10% SDS-polyacrylamide (Tris/glycine) gels and transferred to nitrocellulose membranes (Protran; Whatman, Piscataway, NJ). Membranes were stained with Ponceau S to confirm equal loading (10 or 60 µg/lane), blocked in phosphate-buffered saline (PBS) containing 5% nonfat dry milk, and then probed with antibodies against the FLAG epitope (M2, 1∶1500; Sigma, St. Louis, MO), RGS2 (1∶800; GenWay Biotech Inc., San Diego, CA), GAPDH (1∶1000, Cell Signaling Technology, Danvers, MA ), or calsequestrin (CSQ; 1∶2500; Thermo Scientific, Rockford, IL). After three washes with 0.1% Tween 20-containing PBS, nitrocellulose strips were incubated with appropriate peroxidase-coupled secondary antibodies. Proteins of interest were visualized by chemiluminescence (SuperSignal® West Pico or Femto Substrate; Pierce, Rockford, IL).

### Analysis of mRNA Expression

Total RNA was extracted from snap-frozen ventricular tissue and atrial appendages using TRIzol reagent (Invitrogen, Carlsbad, CA). RNA (0.5 µg) was reverse transcribed into cDNA using TaqMan® Reverse Transcription reagents (Applied Biosystems, Carlsbad, CA). Real-time-PCR (rt-PCR) was performed according to the manufacturer’s instructions using FAM™-labeled TaqMan® probes for RGS2, RGS4, RGS5, RGS6, ANF and 18S and Universal PCR master mix. Each sample was assayed in duplicate in two independent PCR reactions and normalized to 18S expression. Samples without template during rt-PCR served as negative controls. PCR cycling was performed at 95°C for 10 min, followed by 95°C for 15 s and 60°C for 1 min for a total of 40 cycles using ABI Prism 7900. The cycle threshold (C_T_) values corresponding to the PCR cycle number at which fluorescence emission in real time reaches a threshold above the base-line emission were determined using sequence detecting system software (SDS version 2.3; Applied Biosystems). Serial dilutions of cDNA plasmids for rat RGS2 and RGS5 (0.3 to 3x10^6^ copies) were used to confirm linearity of the resulting *C_T_* values.

### Phospholipase C β Activity Assay

Total inositol phosphate (IP) formation was measured as previously described [56]. In brief, freshly dissected atrial and ventricular small tissue pieces (<3 mg) from both sides of the heart were labeled for 90 min with myo-[^3^H]inositol (4 µCi per 24-well; GE Healthcare, Piscataway, NJ) in oxygenated (95% O_2_/5% CO_2_) Krebs Henseleit solution containing (in mM) 118 NaCl, 4.7 KCl, 3 CaCl_2_, 1.2 KH_2_PO_4_, 25 NaHCO_3_, 1.2 MgSO_4_, 0.5 EDTA, and 10 glucose. After addition of LiCl (10 mM final), the tissue pieces were incubated in the presence or absence of endothelin-1 (100 nM) for 30 min at 37°C. Total IP were extracted with 20 mM formic acid, separated by anion exchange chromatography (Dowex AG1-X8), and quantitated by liquid scintillation counting. Data are expressed as cpm/mg tissue, which we previously demonstrated to correlate with normalization to mg protein [56].

### Statistical Analysis

All values are expressed as mean ± SEM for indicated numbers of mice. Group data were compared by one-way or two-way ANOVA (with genotype and surgery type or treatment as variables) followed by Bonferroni post-tests for comparison of replicate means (GraphPad Prism 5). Comparisons between two groups were performed by unpaired two-tailed Student’s t test. A *P* value <0.05 was considered statistically significant.
